# 2-(Adamantan-1-yl)-1,3-bis­(4-methyl­phen­yl)propan-2-ol

**DOI:** 10.1107/S1600536812050647

**Published:** 2012-12-19

**Authors:** Eva Babjaková, Peter Bartoš, Robert Vícha

**Affiliations:** a Department of Chemistry, Faculty of Technology, Tomas Bata University in Zlin, Nám. T. G. Masaryka 275, Zlín, 762 72, Czech Republic; b Department of Chemistry, Faculty of Science, Masaryk University, Kamenice 5, Brno-Bohunice, 625 00, Czech Republic

## Abstract

The conformation of the title compound, C_27_H_34_O, is stabilized by a weak intra­molecular C—H⋯π inter­action. The dihedral angle between the benzene rings is 54.79 (4)°. The adamantane cage consists of three fused cyclo­hexane rings in classical chair conformations, with C—C—C angles in the range 107.75 (10)–111.35 (9)°. Although the mol­ecule contains a hy­droxy group as a conceivable hydrogen-bond donor, this group is sterically hindered by bulky substituents and no hydrogen bonds are observed in the crystal structure.

## Related literature

For the preparation of the title compound, see: Vícha *et al.* (2006 ▶). For other examples of sterically shielded carbinols, see: Babjaková *et al.* (2010 ▶); Vícha & Nečas (2010 ▶). For the structure of a related mol­ecule which does form a hydrogen-bonded dimer in the solid state, see: Vaissermann & Lomas (1997 ▶).
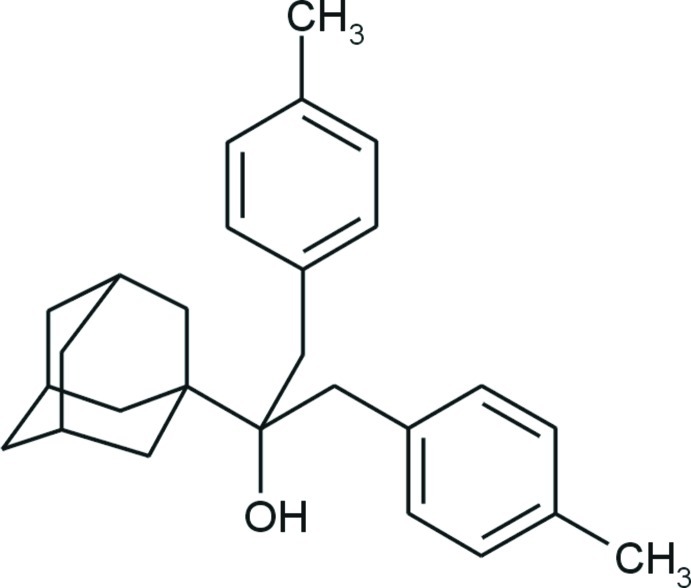


## Experimental

### 

#### Crystal data

C_27_H_34_O
*M* *_r_* = 374.54
Triclinic, 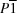

*a* = 6.4065 (2) Å
*b* = 13.1474 (4) Å
*c* = 13.3466 (4) Å
α = 70.718 (3)°
β = 81.700 (3)°
γ = 80.134 (3)°
*V* = 1040.75 (6) Å^3^
*Z* = 2
Mo *K*α radiation
μ = 0.07 mm^−1^
*T* = 120 K
0.50 × 0.50 × 0.40 mm

#### Data collection

Agilent Xcalibur (Sapphire2) diffractometer
Absorption correction: multi-scan (*CrysAlis PRO*; Agilent, 2011 ▶) *T*_min_ = 0.996, *T*_max_ = 1.000
6750 measured reflections
3675 independent reflections
2925 reflections with *I* > 2σ(*I*)
*R*_int_ = 0.010

#### Refinement

*R*[*F* ^2^ > 2σ(*F* ^2^)] = 0.035
*wR*(*F* ^2^) = 0.094
*S* = 1.07
3675 reflections
258 parameters
H atoms treated by a mixture of independent and constrained refinement
Δρ_max_ = 0.18 e Å^−3^
Δρ_min_ = −0.19 e Å^−3^

### 

Data collection: *CrysAlis PRO* (Agilent, 2011 ▶); cell refinement: *CrysAlis PRO*; data reduction: *CrysAlis PRO*; program(s) used to solve structure: *SHELXS97* (Sheldrick, 2008 ▶); program(s) used to refine structure: *SHELXL97* (Sheldrick, 2008 ▶); molecular graphics: *ORTEP-3* (Farrugia, 2012 ▶) and *Mercury* (Macrae *et al.*, 2008 ▶); software used to prepare material for publication: *SHELXL97*.

## Supplementary Material

Crystal structure: contains datablock(s) global, I. DOI: 10.1107/S1600536812050647/nk2194sup1.cif

Structure factors: contains datablock(s) I. DOI: 10.1107/S1600536812050647/nk2194Isup2.hkl

Supplementary material file. DOI: 10.1107/S1600536812050647/nk2194Isup3.cml

Additional supplementary materials:  crystallographic information; 3D view; checkCIF report

## Figures and Tables

**Table 1:** Hydrogen-bond geometry (Å, °) *Cg*1 is the centroid of the C31–C36 ring.

*D*—H⋯*A*	*D*—H	H⋯*A*	*D*⋯*A*	*D*—H⋯*A*
C12—H12⋯*Cg*1	0.94	2.61	3.3577 (12)	136

**Fig. 1.**
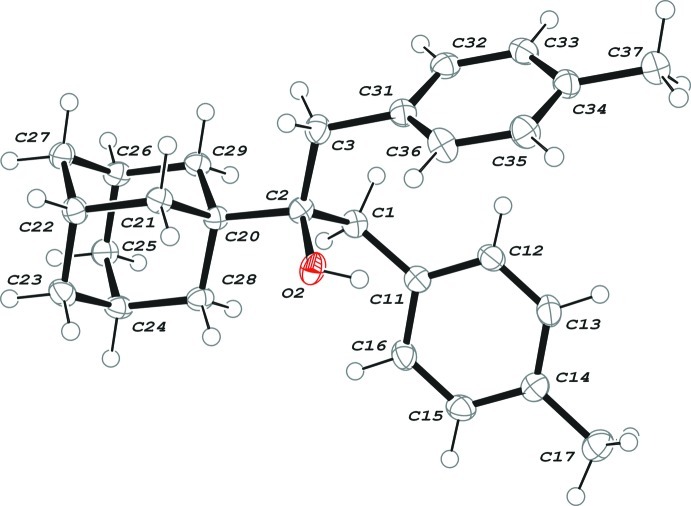
ORTEP of the asymmetric unit with atoms represented as 50% probability ellipsoids. H-atoms are shown as small spheres at arbitrary radii.

**Fig. 2.**
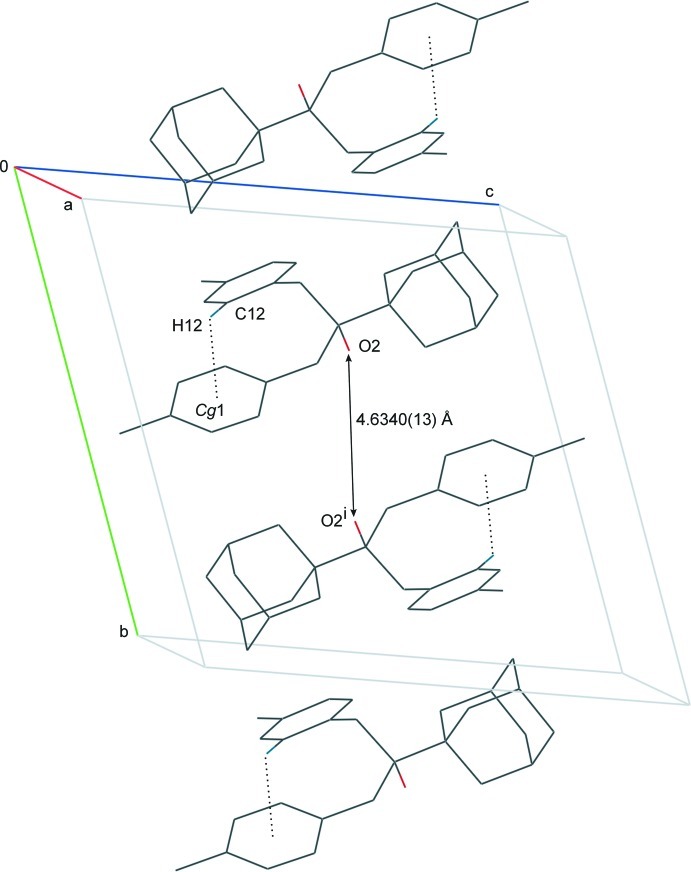
Part of the crystal structure of the title compound showing the intramolecular C—H···π interactions. H-atoms have been omitted for clarity (except for those participating in H-bonds). Symmetry code: (i) -x + 1, -y + 1, -z + 1.

## supplementary crystallographic information

### Comment

The title molecule consists of two *p*-methylated benzene rings, the adamantane cage and propane-2-ol backbone to form a strained tertiary alcohol (Fig. 1). Both benzene rings are essentially planar with maximum deviations from their least squares best planes of 0.0013 (14) Å for C16 and 0.0138 (14) Å for C36, respectively. The dihedral angle between these best planes is 54.79 (4)°. The torsion angles C20—C2—C3—C31, C20—C2—C1—C11, C2—C1—C11—C12, C2—C3—C31—C32 and C21—C20—C2—O2 are -174.97 (10), -129.54 (11), -97.69 (15), 104.62 (14) and 61.95 (12)°, respectively. The conformation of the molecules in the solid state is stabilized by a weak C—H···π interaction, C12—H12···*Cg*1 (*Cg*1 is the centre of gravity of C31–C36), with a C12–*Cg*1 distance of 3.3576 (12) Å (Fig. 2, Table 1). In contrast to the more strained molecules of di(1-adamantyl)(2,5-diisopropylphenyl)methanol those form H-bonded dimers in the solid state (Vaissermann & Lomas, 1997), no H-bonds were observed in the crystal packing of the title compound. The shortest distance between two adjacent O-atoms is 4.6340 (13) Å (Fig. 2). Some other examples of such sterically shielded carbinols have been previously published (Babjaková *et al.*, 2010; Vícha & Nečas, 2010).

### Experimental

The title compound was isolated from complex mixture obtained from the reaction of adamantane-1-carbonyl chloride with 4-methylbenzylmagnesium chloride in diethyl ether as described previously (Vícha *et al.*, 2006). The crystal used for data collection was grown by slow evaporation of an *n*-hexane solution at room temperature.

### Refinement

All carbon bound H atoms were placed at calculated positions and were refined as riding with their *U*_iso_ set to either 1.2*U*_eq_ or 1.5*U*_eq_ (methyl) of the respective carrier atoms; in addition, the methyl H atoms were allowed to rotate about the C—C bond. Oxygen bound H atom was located in a difference Fourier map and refined isotropically with the *U*_iso_ set to 1.5*U*_eq_ of the carrier atom.

### Crystal data

**Table:**

C_27_H_34_O	*Z* = 2
*M**_r_* = 374.54	*F*(000) = 408
Triclinic, *P*1	*D*_x_ = 1.195 Mg m^−^^3^
Hall symbol: -P 1	Melting point: 404 K
*a* = 6.4065 (2) Å	Mo *K*α radiation, λ = 0.71073 Å
*b* = 13.1474 (4) Å	Cell parameters from 4289 reflections
*c* = 13.3466 (4) Å	θ = 3.1–27.8°
α = 70.718 (3)°	µ = 0.07 mm^−^^1^
β = 81.700 (3)°	*T* = 120 K
γ = 80.134 (3)°	Block, colourless
*V* = 1040.75 (6) Å^3^	0.50 × 0.50 × 0.40 mm

### Data collection

**Table:**

Agilent Xcalibur (Sapphire2) diffractometer	3675 independent reflections
Radiation source: Enhance (Mo) X-ray Source	2925 reflections with *I* > 2σ(*I*)
Graphite monochromator	*R*_int_ = 0.010
Detector resolution: 8.4353 pixels mm^-1^	θ_max_ = 25.0°, θ_min_ = 3.2°
ω scan	*h* = −7→7
Absorption correction: multi-scan (*CrysAlis PRO*; Agilent, 2011)	*k* = −15→15
*T*_min_ = 0.996, *T*_max_ = 1.000	*l* = −10→15
6750 measured reflections	

### Refinement

**Table:**

Refinement on *F*^2^	0 restraints
Least-squares matrix: full	H atoms treated by a mixture of independent and constrained refinement
*R*[*F*^2^ > 2σ(*F*^2^)] = 0.035	*w* = 1/[σ^2^(*F*_o_^2^) + (0.0483*P*)^2^ + 0.1419*P*] where *P* = (*F*_o_^2^ + 2*F*_c_^2^)/3
*wR*(*F*^2^) = 0.094	(Δ/σ)_max_ < 0.001
*S* = 1.07	Δρ_max_ = 0.18 e Å^−^^3^
3675 reflections	Δρ_min_ = −0.19 e Å^−^^3^
258 parameters	

### Special details

**Table:**

Experimental. Spectral properties of title compound: ^1^H NMR (500 MHz; CDCl_3_): δ 1.36 (bs, 1H); 1.71 (m, 6H); 1.82(m, 6H); 2.06 (m, 3H); 2.32(s, 6H); 2.68(d, 2H); 3.02(d, 2H); 6.87(d, 4H); 7.01(d, 4H) p.p.m.. ^13^C NMR (75.5 MHz; CDCl_3_): δ 21.2 (CH_3_); 29.0 (CH); 36.7 (CH_2_); 37.4 (CH_2_); 40.0 (CH_2_); 41.3 (C); 76.7(C); 128.9 (CH); 131.2 (CH); 135.5(C); 135.7(C) p.p.m.. IR (KBr): 3581(*s*), 2918(*s*), 2904(*s*), 2879(*s*), 2852(*s*), 1511(*m*), 1454(*m*), 1344(*m*), 1107(w), 1059(w), 1041(w), 997(w), 966(w), 847(w), 818(*m*), 808(*m*), 752(*m*), 714(w), 579(*m*), 496(w), 476(w) cm^-1^ MS (EI, 70 eV): 77 (5), 79 (15), 91 (6), 93 (10), 105 (24), 107 (6), 135 (100), 136 (11), 269 (27), 270 (6) *m/z*(%).
Geometry. All e.s.d.'s (except the e.s.d. in the dihedral angle between two l.s. planes) are estimated using the full covariance matrix. The cell e.s.d.'s are taken into account individually in the estimation of e.s.d.'s in distances, angles and torsion angles; correlations between e.s.d.'s in cell parameters are only used when they are defined by crystal symmetry. An approximate (isotropic) treatment of cell e.s.d.'s is used for estimating e.s.d.'s involving l.s. planes.
Refinement. Refinement of *F*^2^ against ALL reflections. The weighted *R*-factor *wR* and goodness of fit *S* are based on *F*^2^, conventional *R*-factors *R* are based on *F*, with *F* set to zero for negative *F*^2^. The threshold expression of *F*^2^ > 2σ(*F*^2^) is used only for calculating *R*-factors(gt) *etc*. and is not relevant to the choice of reflections for refinement. *R*-factors based on *F*^2^ are statistically about twice as large as those based on *F*, and *R*- factors based on ALL data will be even larger.

### Fractional atomic coordinates and isotropic or equivalent isotropic displacement parameters (Å^2^)

**Table:**

	*x*	*y*	*z*	*U*_iso_*/*U*_eq_	
O2	0.52442 (14)	0.31340 (7)	0.53822 (7)	0.0244 (2)	
H2	0.577 (2)	0.3189 (12)	0.4778 (12)	0.037*	
C1	0.3383 (2)	0.18382 (10)	0.49612 (10)	0.0227 (3)	
H1A	0.3365	0.1115	0.5508	0.027*	
H1B	0.2087	0.1990	0.4584	0.027*	
C2	0.32531 (19)	0.27081 (9)	0.55451 (9)	0.0203 (3)	
C3	0.1531 (2)	0.36778 (10)	0.51005 (9)	0.0246 (3)	
H3A	0.0114	0.3432	0.5345	0.030*	
H3B	0.1616	0.4255	0.5411	0.030*	
C11	0.5301 (2)	0.17801 (9)	0.41677 (10)	0.0214 (3)	
C12	0.5241 (2)	0.22950 (10)	0.30754 (10)	0.0255 (3)	
H12	0.3955	0.2707	0.2810	0.031*	
C13	0.7026 (2)	0.22165 (11)	0.23704 (10)	0.0276 (3)	
H13	0.6933	0.2575	0.1630	0.033*	
C14	0.8939 (2)	0.16309 (10)	0.27152 (10)	0.0242 (3)	
C15	0.9002 (2)	0.11175 (10)	0.38029 (10)	0.0270 (3)	
H15	1.0291	0.0706	0.4066	0.032*	
C16	0.7225 (2)	0.11930 (10)	0.45123 (10)	0.0268 (3)	
H16	0.7323	0.0835	0.5253	0.032*	
C17	1.0884 (2)	0.15472 (12)	0.19488 (11)	0.0322 (3)	
H17A	1.1167	0.2278	0.1504	0.048*	
H17B	1.0641	0.1135	0.1494	0.048*	
H17C	1.2110	0.1174	0.2350	0.048*	
C20	0.28329 (19)	0.21981 (9)	0.67801 (9)	0.0187 (3)	
C21	0.26153 (19)	0.30786 (9)	0.73322 (9)	0.0204 (3)	
H21A	0.3918	0.3439	0.7139	0.025*	
H21B	0.1396	0.3637	0.7077	0.025*	
C22	0.22752 (19)	0.25899 (10)	0.85462 (9)	0.0219 (3)	
H22	0.2142	0.3179	0.8880	0.026*	
C23	0.4157 (2)	0.17335 (10)	0.89566 (10)	0.0249 (3)	
H23A	0.5484	0.2073	0.8777	0.030*	
H23B	0.3933	0.1419	0.9742	0.030*	
C24	0.4346 (2)	0.08420 (10)	0.84410 (10)	0.0233 (3)	
H24	0.5580	0.0282	0.8704	0.028*	
C25	0.2308 (2)	0.03049 (10)	0.87323 (10)	0.0242 (3)	
H25A	0.2069	−0.0018	0.9516	0.029*	
H25B	0.2443	−0.0283	0.8408	0.029*	
C26	0.04286 (19)	0.11655 (10)	0.83190 (10)	0.0225 (3)	
H26	−0.0908	0.0819	0.8512	0.027*	
C27	0.0222 (2)	0.20631 (10)	0.88350 (10)	0.0237 (3)	
H27A	−0.0034	0.1752	0.9619	0.028*	
H27B	−0.1001	0.2618	0.8578	0.028*	
C28	0.47010 (19)	0.13264 (10)	0.72260 (10)	0.0217 (3)	
H28A	0.4849	0.0740	0.6899	0.026*	
H28B	0.6039	0.1657	0.7033	0.026*	
C29	0.07725 (19)	0.16575 (10)	0.71028 (9)	0.0212 (3)	
H29A	−0.0458	0.2206	0.6843	0.025*	
H29B	0.0873	0.1080	0.6768	0.025*	
C31	0.1692 (2)	0.41644 (9)	0.39017 (10)	0.0224 (3)	
C32	0.0303 (2)	0.39663 (10)	0.32966 (10)	0.0245 (3)	
H32	−0.0785	0.3528	0.3646	0.029*	
C33	0.0475 (2)	0.43953 (10)	0.21956 (10)	0.0247 (3)	
H33	−0.0503	0.4251	0.1804	0.030*	
C34	0.2054 (2)	0.50325 (10)	0.16543 (10)	0.0243 (3)	
C35	0.3418 (2)	0.52453 (10)	0.22592 (10)	0.0271 (3)	
H35	0.4506	0.5684	0.1909	0.033*	
C36	0.3228 (2)	0.48324 (10)	0.33613 (10)	0.0265 (3)	
H36	0.4160	0.5008	0.3754	0.032*	
C37	0.2247 (2)	0.55055 (11)	0.04572 (10)	0.0321 (3)	
H37A	0.3466	0.5915	0.0223	0.048*	
H37B	0.0944	0.5992	0.0230	0.048*	
H37C	0.2457	0.4917	0.0140	0.048*	

### Atomic displacement parameters (Å^2^)

**Table:**

	*U*^11^	*U*^22^	*U*^33^	*U*^12^	*U*^13^	*U*^23^
O2	0.0262 (5)	0.0263 (5)	0.0211 (5)	−0.0112 (4)	0.0051 (4)	−0.0072 (4)
C1	0.0260 (7)	0.0196 (6)	0.0224 (7)	−0.0041 (5)	−0.0023 (5)	−0.0058 (5)
C2	0.0207 (6)	0.0182 (6)	0.0213 (6)	−0.0051 (5)	0.0002 (5)	−0.0047 (5)
C3	0.0308 (7)	0.0203 (6)	0.0199 (6)	−0.0006 (5)	−0.0008 (5)	−0.0047 (5)
C11	0.0252 (7)	0.0168 (6)	0.0235 (7)	−0.0021 (5)	−0.0040 (5)	−0.0077 (5)
C12	0.0266 (7)	0.0263 (7)	0.0240 (7)	0.0019 (6)	−0.0073 (6)	−0.0091 (6)
C13	0.0350 (8)	0.0279 (7)	0.0191 (7)	−0.0023 (6)	−0.0027 (6)	−0.0070 (5)
C14	0.0268 (7)	0.0222 (7)	0.0273 (7)	−0.0042 (5)	−0.0011 (5)	−0.0130 (5)
C15	0.0260 (7)	0.0252 (7)	0.0297 (7)	0.0046 (6)	−0.0074 (6)	−0.0107 (6)
C16	0.0342 (8)	0.0233 (7)	0.0200 (7)	0.0026 (6)	−0.0044 (6)	−0.0051 (5)
C17	0.0304 (8)	0.0355 (8)	0.0336 (8)	−0.0048 (6)	0.0016 (6)	−0.0165 (6)
C20	0.0176 (6)	0.0175 (6)	0.0199 (6)	−0.0038 (5)	−0.0005 (5)	−0.0043 (5)
C21	0.0198 (6)	0.0189 (6)	0.0217 (6)	−0.0051 (5)	−0.0016 (5)	−0.0042 (5)
C22	0.0235 (7)	0.0220 (7)	0.0202 (6)	−0.0049 (5)	−0.0021 (5)	−0.0058 (5)
C23	0.0227 (7)	0.0290 (7)	0.0207 (7)	−0.0072 (5)	−0.0041 (5)	−0.0021 (5)
C24	0.0191 (7)	0.0215 (6)	0.0247 (7)	−0.0007 (5)	−0.0039 (5)	−0.0012 (5)
C25	0.0249 (7)	0.0208 (7)	0.0228 (7)	−0.0056 (5)	−0.0001 (5)	−0.0009 (5)
C26	0.0177 (6)	0.0227 (7)	0.0246 (7)	−0.0074 (5)	0.0006 (5)	−0.0029 (5)
C27	0.0216 (7)	0.0261 (7)	0.0195 (6)	−0.0038 (5)	0.0018 (5)	−0.0031 (5)
C28	0.0172 (6)	0.0206 (6)	0.0243 (7)	−0.0037 (5)	0.0001 (5)	−0.0033 (5)
C29	0.0189 (6)	0.0203 (6)	0.0238 (7)	−0.0041 (5)	−0.0031 (5)	−0.0051 (5)
C31	0.0274 (7)	0.0162 (6)	0.0222 (7)	0.0011 (5)	−0.0032 (5)	−0.0061 (5)
C32	0.0227 (7)	0.0196 (6)	0.0279 (7)	−0.0007 (5)	−0.0015 (5)	−0.0044 (5)
C33	0.0248 (7)	0.0242 (7)	0.0263 (7)	0.0003 (5)	−0.0078 (5)	−0.0092 (5)
C34	0.0289 (7)	0.0213 (7)	0.0214 (7)	0.0019 (5)	−0.0040 (5)	−0.0068 (5)
C35	0.0314 (8)	0.0229 (7)	0.0252 (7)	−0.0074 (6)	−0.0030 (6)	−0.0029 (5)
C36	0.0357 (8)	0.0211 (7)	0.0245 (7)	−0.0075 (6)	−0.0077 (6)	−0.0054 (5)
C37	0.0338 (8)	0.0370 (8)	0.0233 (7)	−0.0013 (6)	−0.0040 (6)	−0.0077 (6)

### Geometric parameters (Å, º)

**Table:**

O2—C2	1.4395 (14)	C22—H22	1.0000
O2—H2	0.812 (15)	C23—C24	1.5239 (18)
C1—C11	1.5109 (17)	C23—H23A	0.9900
C1—C2	1.5683 (16)	C23—H23B	0.9900
C1—H1A	0.9900	C24—C25	1.5303 (16)
C1—H1B	0.9900	C24—C28	1.5324 (16)
C2—C3	1.5498 (17)	C24—H24	1.0000
C2—C20	1.5630 (16)	C25—C26	1.5320 (17)
C3—C31	1.5096 (16)	C25—H25A	0.9900
C3—H3A	0.9900	C25—H25B	0.9900
C3—H3B	0.9900	C26—C27	1.5301 (17)
C11—C16	1.3894 (17)	C26—C29	1.5342 (16)
C11—C12	1.3930 (17)	C26—H26	1.0000
C12—C13	1.3841 (18)	C27—H27A	0.9900
C12—H12	0.9500	C27—H27B	0.9900
C13—C14	1.3828 (18)	C28—H28A	0.9900
C13—H13	0.9500	C28—H28B	0.9900
C14—C15	1.3875 (18)	C29—H29A	0.9900
C14—C17	1.5071 (17)	C29—H29B	0.9900
C15—C16	1.3836 (18)	C31—C36	1.3900 (17)
C15—H15	0.9500	C31—C32	1.3920 (18)
C16—H16	0.9500	C32—C33	1.3845 (17)
C17—H17A	0.9800	C32—H32	0.9500
C17—H17B	0.9800	C33—C34	1.3881 (18)
C17—H17C	0.9800	C33—H33	0.9500
C20—C21	1.5430 (16)	C34—C35	1.3891 (18)
C20—C28	1.5444 (17)	C34—C37	1.5059 (17)
C20—C29	1.5454 (16)	C35—C36	1.3845 (17)
C21—C22	1.5311 (16)	C35—H35	0.9500
C21—H21A	0.9900	C36—H36	0.9500
C21—H21B	0.9900	C37—H37A	0.9800
C22—C23	1.5293 (17)	C37—H37B	0.9800
C22—C27	1.5325 (17)	C37—H37C	0.9800
			
C2—O2—H2	108.8 (11)	C24—C23—H23B	109.9
C11—C1—C2	116.07 (10)	C22—C23—H23B	109.9
C11—C1—H1A	108.3	H23A—C23—H23B	108.3
C2—C1—H1A	108.3	C23—C24—C25	109.95 (10)
C11—C1—H1B	108.3	C23—C24—C28	109.94 (10)
C2—C1—H1B	108.3	C25—C24—C28	109.14 (10)
H1A—C1—H1B	107.4	C23—C24—H24	109.3
O2—C2—C3	107.41 (9)	C25—C24—H24	109.3
O2—C2—C20	105.06 (9)	C28—C24—H24	109.3
C3—C2—C20	111.35 (10)	C24—C25—C26	108.95 (10)
O2—C2—C1	111.17 (9)	C24—C25—H25A	109.9
C3—C2—C1	110.22 (10)	C26—C25—H25A	109.9
C20—C2—C1	111.46 (9)	C24—C25—H25B	109.9
C31—C3—C2	115.30 (10)	C26—C25—H25B	109.9
C31—C3—H3A	108.4	H25A—C25—H25B	108.3
C2—C3—H3A	108.4	C27—C26—C25	109.41 (10)
C31—C3—H3B	108.4	C27—C26—C29	109.57 (10)
C2—C3—H3B	108.4	C25—C26—C29	110.20 (10)
H3A—C3—H3B	107.5	C27—C26—H26	109.2
C16—C11—C12	117.03 (11)	C25—C26—H26	109.2
C16—C11—C1	120.32 (11)	C29—C26—H26	109.2
C12—C11—C1	122.66 (11)	C26—C27—C22	109.25 (10)
C13—C12—C11	121.09 (12)	C26—C27—H27A	109.8
C13—C12—H12	119.5	C22—C27—H27A	109.8
C11—C12—H12	119.5	C26—C27—H27B	109.8
C14—C13—C12	121.78 (12)	C22—C27—H27B	109.8
C14—C13—H13	119.1	H27A—C27—H27B	108.3
C12—C13—H13	119.1	C24—C28—C20	111.27 (10)
C13—C14—C15	117.24 (12)	C24—C28—H28A	109.4
C13—C14—C17	121.84 (12)	C20—C28—H28A	109.4
C15—C14—C17	120.92 (12)	C24—C28—H28B	109.4
C16—C15—C14	121.29 (12)	C20—C28—H28B	109.4
C16—C15—H15	119.4	H28A—C28—H28B	108.0
C14—C15—H15	119.4	C26—C29—C20	110.41 (10)
C15—C16—C11	121.57 (12)	C26—C29—H29A	109.6
C15—C16—H16	119.2	C20—C29—H29A	109.6
C11—C16—H16	119.2	C26—C29—H29B	109.6
C14—C17—H17A	109.5	C20—C29—H29B	109.6
C14—C17—H17B	109.5	H29A—C29—H29B	108.1
H17A—C17—H17B	109.5	C36—C31—C32	117.51 (11)
C14—C17—H17C	109.5	C36—C31—C3	121.18 (11)
H17A—C17—H17C	109.5	C32—C31—C3	121.31 (11)
H17B—C17—H17C	109.5	C33—C32—C31	121.35 (12)
C21—C20—C28	107.74 (10)	C33—C32—H32	119.3
C21—C20—C29	107.66 (10)	C31—C32—H32	119.3
C28—C20—C29	107.99 (9)	C32—C33—C34	121.08 (12)
C21—C20—C2	110.48 (9)	C32—C33—H33	119.5
C28—C20—C2	110.59 (9)	C34—C33—H33	119.5
C29—C20—C2	112.22 (9)	C33—C34—C35	117.56 (11)
C22—C21—C20	111.34 (9)	C33—C34—C37	121.41 (12)
C22—C21—H21A	109.4	C35—C34—C37	121.00 (12)
C20—C21—H21A	109.4	C36—C35—C34	121.48 (12)
C22—C21—H21B	109.4	C36—C35—H35	119.3
C20—C21—H21B	109.4	C34—C35—H35	119.3
H21A—C21—H21B	108.0	C35—C36—C31	120.96 (12)
C23—C22—C21	110.08 (10)	C35—C36—H36	119.5
C23—C22—C27	109.69 (10)	C31—C36—H36	119.5
C21—C22—C27	108.76 (10)	C34—C37—H37A	109.5
C23—C22—H22	109.4	C34—C37—H37B	109.5
C21—C22—H22	109.4	H37A—C37—H37B	109.5
C27—C22—H22	109.4	C34—C37—H37C	109.5
C24—C23—C22	108.95 (10)	H37A—C37—H37C	109.5
C24—C23—H23A	109.9	H37B—C37—H37C	109.5
C22—C23—H23A	109.9		
			
C11—C1—C2—O2	−12.69 (14)	C27—C22—C23—C24	60.05 (13)
C11—C1—C2—C3	106.29 (12)	C22—C23—C24—C25	−60.43 (12)
C11—C1—C2—C20	−129.54 (11)	C22—C23—C24—C28	59.76 (13)
O2—C2—C3—C31	70.51 (13)	C23—C24—C25—C26	60.58 (13)
C20—C2—C3—C31	−174.97 (10)	C28—C24—C25—C26	−60.10 (13)
C1—C2—C3—C31	−50.74 (14)	C24—C25—C26—C27	−60.16 (12)
C2—C1—C11—C16	82.61 (14)	C24—C25—C26—C29	60.37 (13)
C2—C1—C11—C12	−97.68 (14)	C25—C26—C27—C22	60.14 (12)
C16—C11—C12—C13	0.28 (18)	C29—C26—C27—C22	−60.78 (13)
C1—C11—C12—C13	−179.44 (12)	C23—C22—C27—C26	−60.16 (13)
C11—C12—C13—C14	−0.2 (2)	C21—C22—C27—C26	60.29 (12)
C12—C13—C14—C15	0.21 (19)	C23—C24—C28—C20	−60.11 (13)
C12—C13—C14—C17	−179.83 (12)	C25—C24—C28—C20	60.57 (13)
C13—C14—C15—C16	−0.25 (19)	C21—C20—C28—C24	57.46 (12)
C17—C14—C15—C16	179.79 (12)	C29—C20—C28—C24	−58.57 (13)
C14—C15—C16—C11	0.3 (2)	C2—C20—C28—C24	178.29 (9)
C12—C11—C16—C15	−0.32 (18)	C27—C26—C29—C20	60.53 (13)
C1—C11—C16—C15	179.40 (12)	C25—C26—C29—C20	−59.90 (13)
O2—C2—C20—C21	61.95 (12)	C21—C20—C29—C26	−58.42 (12)
C3—C2—C20—C21	−54.02 (13)	C28—C20—C29—C26	57.66 (13)
C1—C2—C20—C21	−177.55 (10)	C2—C20—C29—C26	179.80 (9)
O2—C2—C20—C28	−57.24 (12)	C2—C3—C31—C36	−75.74 (15)
C3—C2—C20—C28	−173.21 (10)	C2—C3—C31—C32	104.61 (14)
C1—C2—C20—C28	63.26 (12)	C36—C31—C32—C33	1.60 (18)
O2—C2—C20—C29	−177.90 (9)	C3—C31—C32—C33	−178.75 (11)
C3—C2—C20—C29	66.14 (13)	C31—C32—C33—C34	0.53 (19)
C1—C2—C20—C29	−57.40 (13)	C32—C33—C34—C35	−1.57 (19)
C28—C20—C21—C22	−57.24 (12)	C32—C33—C34—C37	−179.77 (12)
C29—C20—C21—C22	59.00 (12)	C33—C34—C35—C36	0.49 (19)
C2—C20—C21—C22	−178.14 (9)	C37—C34—C35—C36	178.69 (12)
C20—C21—C22—C23	59.62 (13)	C34—C35—C36—C31	1.7 (2)
C20—C21—C22—C27	−60.58 (13)	C32—C31—C36—C35	−2.67 (18)
C21—C22—C23—C24	−59.59 (12)	C3—C31—C36—C35	177.67 (11)

### Hydrogen-bond geometry (Å, º)

*Cg*1 is the centroid of the C31–C36 ring.

**Table:**

*D*—H···*A*	*D*—H	H···*A*	*D*···*A*	*D*—H···*A*
C12—H12···*Cg*1	0.94	2.61	3.3577 (12)	136
